# Chemical Biology Strategies to Study Autophagy

**DOI:** 10.3389/fcell.2018.00160

**Published:** 2018-11-27

**Authors:** Piyush Mishra, Veena Ammanathan, Ravi Manjithaya

**Affiliations:** Autophagy Lab, Molecular Biology and Genetics Unit, Jawaharlal Nehru Centre for Advanced Scientific Research, Bengaluru, India

**Keywords:** autophagy, high throughput, chemical biology, luciferase, small molecule screening, fluorescence microscopy

## Abstract

Growing amount of evidence in the last two decades highlight that macroautophagy (generally referred to as autophagy) is not only indispensable for survival in yeast but also equally important to maintain cellular quality control in higher eukaryotes as well. Importantly, dysfunctional autophagy has been explicitly shown to be involved in various physiological and pathological conditions such as cell death, cancer, neurodegenerative, and other diseases. Therefore, modulation and regulation of the autophagy pathway has emerged as an alternative strategy for the treatment of various disease conditions in the recent years. Several studies have shown genetic or pharmacological modulation of autophagy to be effective in treating cancer, clearing intracellular aggregates and pathogens. Understanding and controlling the autophagic flux, either through a genetic or pharmacological approach is therefore a highly promising approach and of great scientific interest as spatiotemporal and cell-tissue-organ level autophagy regulation is not clearly understood. Indeed, chemical biology approaches that identify small molecule effectors of autophagy have thus a dual benefit: the modulators act as tools to study and understand the process of autophagy, and may also have therapeutic potential. In this review, we discuss different strategies that have appeared to screen and identify potent small molecule modulators of autophagy.

## Introduction

Macroautophagy (herein autophagy) is a major intracellular process that is critically crucial for maintaining cellular homeostasis. Autophagy has been reported in several organisms from different kingdoms ranging from yeast to humans suggesting that it is an evolutionarily conserved process. This process was first reported by Christian de Duve (Deter et al., 1967), when he observed organelles captured within the lysosomes with the help of electron microscopy (De Duve and Wattiaux, 1966). This entire phenomenon of cargo capture and ultimately its degradation in the lysosomes is called “autophagic flux" (Klionsky, 2007; Rabinowitz and White, 2010; Boya et al., 2013).

Basal levels of autophagy occur in all cells during nutrient rich conditions and help in housekeeping functions to maintain cellular quality control by clearance of damaged or surplus organelles and misfolded proteins, recycling and providing basic building blocks like amino acids for reuse (Mizushima and Klionsky, 2007; Musiwaro et al., 2013). However, the levels of autophagy are highly modulated in response to different stimulus, both intracellular and exogenous such as starvation, pathogen invasion, organelle damage and protein aggregation in cytoplasm (Takeshige et al., 1992; Komatsu and Ichimura, 2010). Because autophagy is central to maintaining cellular homeostasis, defective autophagy has been attributed to a variety of disease conditions such as cardiovascular diseases, atherosclerosis, certain myopathies, innate and adaptive immune responses, neurodegeneration and cancer (Choy and Roy, 2013; Kroemer, 2015).

**FIGURE 1:**
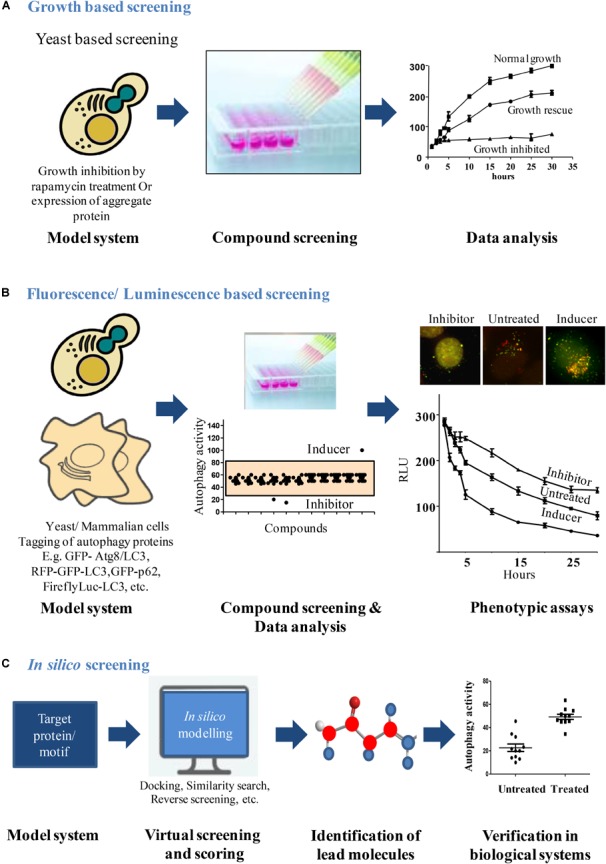
Workflow for screening autophagy modulators. **(A)** Growth based screening: growth inhibition can be induced in yeast due to over expression of aggregate proteins or rapamycin treatment. This cytostatic effect exhibited by yeast can be used as a platform to screen compounds that rescue the growth lag through autophagy induction. After compound treatments, analysis of yeast growth curves identifies the compounds that rescued the growth lag. **(B)** Fluorescence/Luminescence based screening: fluorescent or luminescent reporters are tagged to autophagy proteins for transfection in yeast or mammalian model systems. Modulators of autophagy from chemical libraries are obtained by analyzing the fluorescent/luminescent signal intensities or by visualizing the autophagic vesicle formation by microscopy. **(C)** *In silico* screening: structures of autophagy proteins/motifs of interest can be obtained from data sources like Protein Data Bank and can be used as a model system to identify chemical molecules that bind using *in silico* modeling softwares. The selected lead molecules are then verified in biological system to validate its ability to modulate the process.

Dysfunction of autophagy leads to cell death, cancer, neurodegenerative, and other diseases. Therefore, studying the molecular aspects of autophagy is of current research interest for the treatment of various disease conditions. Genetic and pharmacological modulation of autophagy has been shown to be beneficial in many such situations (Rubinsztein et al., 2012). Modulation of autophagy has been shown to be beneficial in diseases such as diabetes, cancers, neurodegenerative disorders and some infectious diseases (Sarkar et al., 2007; Sarkar and Rubinsztein, 2008). Several studies in the recent years have discovered novel or repurposed drugs for restoring autophagic balance. For instance, Rapamycin, an autophagy inducer and its analogs were used by Ravikumar et al., to abrogate neurodegeneration in a *Drosophila* based model by enhancing the rates of autophagy (Ravikumar et al., 2004; Sarkar, 2013a). In some of these studies, distinct assays have been developed and used for a High Throughput Screening (HTS) to identify small molecules that modulate autophagy (Table 1). Several autophagy modulators have been discovered in the recent past but very few of them have led to potential candidate drug molecules. Many of these compounds are specific toward different targets in the autophagy pathway. For example, specific screens to identify novel candidate molecules such as ULK1 (Rosenberg et al., 2015), ATG4 (Ketteler and Seed, 2008), class III phosphatidylinositol 3-kinase (Farkas et al., 2011), and MTOR (Butcher et al., 2006), have been carried out. In addition, compounds with broad spectrum effects have also been identified as well (Sarkar, 2013b). The scope for the discovery of new autophagy modulators that can be later taken up to clinical trials is ever increasing. It has been postulated that deeper insights into autophagy through chemical modulation can lead to better understanding of various diseases. In addition, understanding of the mechanism of these molecules may provide deeper mechanistic insights and understanding of the finely regulated process of autophagy. Chemical biology approach to study autophagy can be compared to a genetic screen (Tsukada and Ohsumi, 1993; Thumm et al., 1994; Harding et al., 1995; Titorenko et al., 1995), where further studies on the hits reveal more about the mechanism of autophagy. For example, just as the identification of a gene and its function, a manner in which a small molecule modulates autophagy can also shed some light regarding the regulation of autophagy (Seglen and Gordon, 1982; Kunz et al., 1993). In search of potential candidate drugs that moderate autophagy, identifying small molecule modulators of autophagy is the primary step. Small molecule study will further enhance the understanding of autophagy and related pathways. Thus, having a robust, sensitive assay to monitor autophagic flux that could be performed at a high throughput rate for the purpose of screening modulators of autophagy is of primary importance (Figure 1). In this review, we discuss some of the pharmacological strategies undertaken in the recent past to identify novel autophagy modulators (Table 2).

**Table 1:** Autophagy modulators identified through High Throughput Screening of Chemical compound libraries.

Compound	Autophagy	General/Selective	Mechanism of	Reference
name	modulation	autophagy modulator	autophagy modulation	
ARP101	Inducer	General	Induction of autophagosome biogenesis	Jo et al., 2011
Bay 11	Inhibitor	General	Inhibition of autophagosome biogenesis	Mishra et al., 2017a
BRD5631	Inducer	Aggrephagy/Xenophagy	–	Kuo et al., 2015
Carbamazepine	Inducer	Xenophagy	By myo-inositol depletion and AMPK activation	Schiebler et al., 2015
Cardiac glycosides, e.g., Digoxin, Helveticoside	Inducer	General	Inhibition of Na^+^K^+^ATPases leading to increase in Ca^2+^ levels	Hundeshagen et al., 2011
KU55933 and Gö6976	Inhibitor	General	Inhibition of PI3K	Farkas et al., 2011
Loperamide	Inducer	Aggrephagy	Regulation of intracellular Ca^2+^ levels	Zhang et al., 2007
P29A03	Inducer	General	Increase in Beclin levels	Lee et al., 2013
P23C07	Inhibitor	General	Inhibition of autophagosomes fusion with lysosomes	Lee et al., 2013
Rottlerin	Inducer	General	Inhibition of mTOR through TSC2 pathway	Balgi et al., 2009
6-Bio	Inducer	Aggrephagy	GSK-3 beta inhibitor	Suresh et al., 2017
Fasudil	Inducer	General	–	Iorio et al., 2010
Flubendazole	Inducer	Xenophagy	Microtubules destabiliser	Chauhan et al., 2015
Minoxidil and clonidine	Inducer	Aggrephagy	Modulation of cAMP levels	Williams et al., 2008
Niclosamide	Inducer	General	Inhibition of mTOR	Balgi et al., 2009
Perhexiline	Inducer	General	Inhibition of mTOR	Balgi et al., 2009
SEN177	Inducer	Aggrephagy	Inhibition of glutaminyl cyclase	Jimenez-Sanchez et al., 2015
SMER10, SMER18, SMER28	Inducer	Aggrephagy	–	Sarkar et al., 2007
Trifluoperazine	Inducer	Aggrephagy/Xenophagy	Increase in FYVE containing vesicles	Zhang et al., 2007
Tamoxifen	Inducer	Xenophagy	Estrogen and G protein coupled receptor GPR30 antagonist shown to inhibit intracellular *Toxoplasma*	Dittmar et al., 2016
Valproic acid	Inducer	Xenophagy	By myo-inositol depletion and AMPK activation	Schiebler et al., 2015
XCT 790	Inducer	Aggrephagy/Xenophagy	ERR alpha inhibitor	Suresh et al., 2018
ZPCK	Inhibitor	General	Inhibition of cargo degradation within lysosomes	Mishra et al., 2017a

**Table 2:** Summary of HTS assays for compound libraries.

Model	Assay principle	Read out	Compound(s)	Autophagy	Reference
system			identified	modulation	
Yeast	Rescue of rapamycin induced growth inhibition	Growth based assay	LY-83583	–	Butcher et al., 2006
	Rescue of rapamycin induced growth inhibition	Growth based assay	SMER 10, 18, and 28	Inducer	Sarkar et al., 2007
	Rescue of rapamycin induced growth inhibition	Growth based assay	SMIRs	Inhibitor	Sarkar et al., 2007
	Rescue of SNCA α-synuclein induced growth lag	Growth based assay	6-Bio	Inducer	Suresh et al., 2017
	Degradation of luciferase tagged peroxisomes	Luminescence	Bay11, ZPCK	Inhibitor	Mishra et al., 2017a
Mammalian cells	Increase in number of autophagosomes (GFP-LC3)	Fluorescence microscopy	ARP101	Inducer	Jo et al., 2011
	Increase in number of autophagosomes and autolysosomes (mCherry-GFP-LC3)	Flow cytometry	Cardiac glycosides	Inducer	Hundeshagen et al., 2011
	Degradation of autophagy adaptor proteins (GFP-p62, GFP-NBR1)	Flow cytometry	Lactacystin	Inhibitor	Larsen et al., 2010
	Reduction in intracellular *Mycobacterium tubercluosis*	Fluorescence microscopy	Valproic acid	Inducer	Schiebler et al., 2015
	Increase in autophagosomes and autolysosomes (mCherry-GFP-LC3)	High-content fluorescent microscopy	Flubendazole	Inducer	Chauhan et al., 2015
	Degradation of lipid droplets	Fluorescence microscopy	P23C07	Inhibitor	Lee et al., 2013
	Ratio of GFP-LC3 (autophagosomes) and cytosolic RFP-LC3ΔG (internal control) using the probe GFP-LC3-RFP-LC3ΔG	High-content fluorescent microscopy and flow cytometry	Deslanoside, Cladribine	Inducer	Kaizuka et al., 2016
	Ratio of GFP-LC3 (autophagosomes) and cytosolic RFP-LC3ΔG (internal control) using the probe GFP-LC3-RFP-LC3ΔG	High-content fluorescent microscopy and flow cytometry	Mebendazole Nelarabine	Inhibitor	Kaizuka et al., 2016
	Clearance of A30P α-synuclein	Fluorescence microscopy	Minoxidil and clonidine	Inducer	Williams et al., 2008
	Quantitation of Nuclear LC3	High-content fluorescent microscopy	NSC179818, NSC60785	–	Kolla et al., 2018
	Degradation of luciferase tagged adaptor protein (Luc2p-p62 and Luc2p-p62ΔU)	Luminescence	Temozolomide	Inducer	Min et al., 2018
	Renilla Luc tagged LC3 turnover	Luminescence	KU55933 and Gö6976	Inhibitor	Farkas et al., 2011
	Comparison of data expression pattern	*In silico* data mining	Fasudil	Inducer	Iorio et al., 2010

## Conventional Autophagy Assays

The real time analysis of autophagy in cells tissues principally been performed via qualitative measures. These assays identify autophagosomes or measure the conversion of LC3I to LC3II (Atg8 in yeast) either through western blotting or microscopy (Klionsky et al., 2016). Owing to the conserved nature of autophagy (Mizushima et al., 1998; Kabeya et al., 2000; Meijer et al., 2007), the use of yeast as a model system to study autophagy is still widely recognized, even after the identification of homologous Atg sequences in mammalian cells. This is primarily because of the ease of handling and the vast array of biochemical and genetic tools available to carry out autophagy studies. Several different techniques to monitor autophagy are well established in yeast (Torggler et al., 2017). For example, Pho8Δ60 assay provides readout for bulk autophagy (Noda et al., 1995). Wild type alkaline phosphatase protein moves from ER (inactive) to vacuole where it gets activated. Deletion of first 60 amino acids from the N-terminal makes the mutated protein cytosolic which is taken up by the autophagosome machinery along with other cytosolic contents and delivered to vacuole for bulk degradation. The action of vacuolar proteases activates the Pho8Δ60, which can act on different substrates to dephosphorylate them. Depending on the substrate being used, the readout could be measured using either photometry or fluorimetry.

Other classical assays in yeast include monitoring the degradation of fluorescent tagged Atg8 (GFP-Atg8), either through microscopy or immunoblotting (Kirisako et al., 1999; Suzuki et al., 2001; Meiling-Wesse et al., 2002). Similarly, autophagic degradation of certain different cargoes like PGK1 or radiolabeled long-lived proteins and organelles like peroxisomes (discussed in later sections) and mitochondria can be chased (Tsukada and Ohsumi, 1993; Kissova et al., 2004; Sakai et al., 2006; Welter et al., 2010; Motley et al., 2012).

Although yeast studies provide a reliable and efficient way to study autophagy, considering the complexity in higher eukaryotes, the results cannot be always extrapolated. Keeping the role of autophagy in different physiological and pathological contexts in mind, several different autophagy assays have been developed in cell culture (Tooze et al., 2015; Orhon and Reggiori, 2017). Many of these assays rely on the status of LC3B protein, which is a mammalian homolog of yeast Atg8 protein and is involved in biogenesis and maturation of autophagosome (Kabeya et al., 2000; Mizushima and Yoshimori, 2007; Weidberg et al., 2010; Nguyen et al., 2016). LC3 gets conjugated to phosphatidyl ethanolamine (PE) on the autophagosome membrane and is the sole marker for autophagosomes right from the biogenesis to its degradation. The form of LC3B conjugated to PE is called LC3B II, while the cytosolic, unconjugated form is referred to as LC3B I. This led to development of various LC3 based assays for monitoring the autophagic flux. Other autophagy marker protein widely utilized for the purpose of autophagy assays is p62/SQSTM1, which is an adaptor protein that helps in cargo sequestration (Bjorkoy et al., 2005). Different fluorescent reporters are tagged to these markers (mRFP/GFP-LC3) to visualize them under the microscope (Kabeya et al., 2000). *In vivo* studies have also been conducted in the past using the fluorescently labeled LC3 marker. Mizushima et al., used a transgenic mice model expressing the GFP-LC3 protein to show that autophagy occurs in all the cell types. The basal levels of autophagy vary in different tissues and starvation stimulus induces autophagy over and above the basal levels in all the tissues (Mizushima et al., 2004). Tandem fluorescent tags on these proteins (mRFP/mCherry-GFP-LC3) provide an added benefit of visualizing different stages of autophagic flux (Kimura et al., 2007). This reporter is an indicator of conversion of autophagosomes into autolysosomes upon fusion with lysosomes, wherein the autophagosomes emit both mRFP and GFP signals (mRFP^+^ GFP^+^) whereas the autolysosomes emit only mRFP signal (mRFP^+^ GFP^-^) because GFP is acid-labile and is quenched in the acidic environment of the autolysosomes.

The cytoplasmic autophagic flux of proteins is too small to be chased over a time course using an assay (Welter et al., 2010). The turnover rate of cytosolic proteins through basal autophagy is less and does not provide a broad window or physiological range to carry out a screen using protein degradation as a measure. In turn, having an inducible cargo that is specifically degraded through autophagy provides a higher working range. The inadaptability of the conventional autophagy assays into a high throughput setting presents a major limitation and hence makes the small molecule screening a very cumbersome process (Cheong and Klionsky, 2008; Wang and Subramani, 2017).

## High Throughput Assays to Monitor Autophagy

Multiple aspects and steps of the autophagy pathway have been exploited to establish several different HTS assay systems both in yeast and mammalian cells. These have also led to identification of potent novel autophagy modulators (Figure 1). Studies on these modulators have not only revealed their therapeutic potential but led to better understanding of the autophagy process.

### Growth Based Autophagy Assays

MTOR is a nutrient sensor and hence is central to cells growth. MTOR also is a regulator of the autophagy pathway (Noda and Ohsumi, 1998; Loewith and Hall, 2011). Rapamycin, an inhibitor of MTOR, activates autophagy pathway (Abraham and Wiederrecht, 1996). This understanding has been widely utilized to develop assays to monitor autophagy via MTOR activity. Butcher *et al.*, developed an assay that monitored the growth of yeast cells each harboring a different plasmid from a pool of 3900 overexpression plasmids in the presence of rapamycin, which is an inhibitor of MTOR (Butcher et al., 2006). Yeast cells when cultured in the presence of rapamycin, undergo growth inhibition, because of block in TOR pathway. From the pool of overexpression plasmids, candidate gene products were identified that suppressed the cytostatic effect of rapamycin and were involved in the TOR pathway. They also characterized the mechanism of LY-83583. LY-83583 is a novel molecule that suppressed the rapamycin-induced growth inhibition and its several candidate targets were also implicated. Sarkar et al. (2007), used yeast to identify small molecule enhancers (SMERs) and inhibitors (SMIRs) of rapamycin using the same strategy. From the screening, 21 SMIRs and 12 SMERs were listed that were structurally non-redundant. They identified SMERs that could enhance autophagy independently of MTOR, and these SMERs (SMER 10, 18, and 28) when tested in mouse and Drosophila models decreased the toxicity associated with mutant Huntington protein, also reflecting on the therapeutic potential of these compounds (Sarkar et al., 2007). The HTS utilized a chemical genetic suppressor platform to rescue or elevate the growth inhibitory properties of rapamycin on wild type yeast cells (Huang et al., 2004). Therefore, because of the involvement of MTOR pathway in regulating autophagy, a simple screen based on the growth of yeast was able to give therapeutically potent small molecule hits.

A growth-based neurotoxicity assay in yeast was also utilized by Suresh et al., to identify novel autophagy enhancer 6-Bio that ameliorates α-synuclein toxicity. The compound 6-Bio effectively cleared toxic aggregates in an autophagy dependent manner in both yeast as well as mammalian cells. More importantly, the action of the compound was conserved and showed neuroprotection in a pre-clinical mouse model of Parkinson’s disease (Suresh et al., 2017).

### Fluorescence Based High Throughput Assays

Fluorescence based microscopy assays are the most commonly used techniques to monitor autophagic flux. Autophagy, being a multistep process involving several molecular players, presents with a number of markers that can be tagged with a fluorescent probe and the autophagy rates can be monitored. Interestingly, this has also been exploited to design several high content-based imaging strategies to screen for novel autophagy modulators (Figure 1).

Clearance of toxic poly glutamine aggregates in cell culture was also demonstrated by using autophagy enhancers obtained from an image based HTS of GFP tagged LC3 puncta representing autophagosomes (Zhang et al., 2007). The number, size and intensity of the autophagosomes were analyzed and quantified using high throughput fluorescence microscopy. GFP-LC3 was used as a probe in an automated microscopy cell-based assay to identify chemical enhancers that rapidly led to an increase in autophagosome content (Balgi et al., 2009). The same reporter was also used by Jo et al. (2011), to identify ARP101, that inhibits matrix metalloproteinase-2 (MMP-2) selectively; as an inducer of autophagy- associated cell death in cancer cells. A high content, flow cytometry based screening approach was used to screen Prestwick Chemical Library containing FDA approved drugs by looking at autolysosome formation and degradation and also endolysosomal activities under basal and stimulated autophagy conditions (Hundeshagen et al., 2011). This study used three different probes to investigate different stages of autophagic flux (GFP-LC3 for autophagosome, mCherry-GFP-LC3 for autophagosome and autolysosome) and endocytic activity (GFP-Rab7). From the screening, cardiac glycosides were validated as potent enhancers of autophagic flux. The same GFP-LC3 probe has also been used by Kuo et al. (2015), to screen 59,541 small molecules prepared by stereoselective diversity-oriented chemical synthesis and identification of enhancers of autophagy.

Larsen et al. (2010), followed the degradation of three fluorescent tagged autophagy markers: GFP-p62, GFP-NBR1, or GFP-LC3 by flow cytometry of live cells after their promoter has been turned off. Relative degradation rates of these three promoters was analyzed under basal autophagy conditions. Through single cell analysis, GFP-LC3B was found to be the most stable protein whereas GFP-NBR1 was the reporter that was most effectively degraded. The degradation of GFP-p62 was observed to show the strongest response to nutrient limitation condition and was reported to be the best reporter out of the three. Chemical screening strategies have also been used to identify novel target processes that activate autophagy (Chauhan et al., 2015). In this study, LC3B puncta in HeLa cells stably expressing mRFP-GFP-LC3B were analyzed using high-content (HC) image analysis of and revealed a novel role of microtubules, which when altered resulted in autophagy induction.

Autophagy dependent degradation of lipid droplets (LDs) was also used for the development of a high content screening platform to discover novel autophagy modulators (Lee et al., 2013). In this study, an indolizine-based fluorescent skeleton called Seoul-Fluor (SF) (Kim et al., 2011) that stains the hydrophobic LDs was used and its subsequent degradation via autophagy was followed.

Two anticonvulsants were discovered as mTOR independent autophagy enhancers, from a functional cell-based screening of FDA-approved drugs that were further shown to clear intracellular population of *Mycobacterium tuberculosis* (Schiebler et al., 2015). In this screen, a library of 214 compounds was screened for its ability to kill intracellular luminescent strain of *M. bovis* BCG (bacille-Calmette-Guerin, live attenuated strain of *M. bovis*) within macrophages. These hits were further validated both in primary macrophages and autophagy null cells and also for their effect on autophagy in an mTOR independent manner. The probe GFP-LC3-RFP-LC3ΔG developed by Kaizuka et al. (2016), serves as a cumulative index for autophagy activity. The probe utilizes the protease activity of the ATG4 family of proteins. Upon cleavage of the fusion protein by ATG4, GFP-LC3 gets associated with the autophagosomes and then degraded upon subsequent fusion to lysosomes. RFP-LC3ΔG on the other hand is cytosolic due to the deletion of glycine at the C-terminal of LC3. This probe can be utilized in different settings like high throughput microscopy, flow cytometry and microplate readers and is also amenable to screening small molecule modulators of autophagy by comparing the ratio of GFP/RFP.

A high content screening in HeLa cells using EGFP-LC3 reporter identified several autophagy inhibitors. These compounds were then further analyzed using an array of phenotypic cell-based assays. The screening strategy identified several hitherto unknown target proteins amongst the well defined targets like Vps34 and ULK1 (Peppard et al., 2014). In a first of a kind, a high content screening using the fluorescent LC3 reporter, a library of 1539 chemical compounds was aimed to identify modulators that affected the nuclear localization of LC3. Potent modulators were identified that may help in the understanding of LC3 nuclear-cytoplasmic localization (Kolla et al., 2018).

Parkinson’s disease associated protein A30P α-synuclein is a substrate for autophagy and has been used to study aggregate clearance by autophagy in the past. One such study used A30P α-synuclein clearance by autophagy as a primary screen to identify novel autophagy enhancers. Using this screen L-type Ca^2+^ channel antagonists, the K^+^_ATP_ channel opener minoxidil, and the G_i_ signaling activator clonidine were identified as autophagy inducers that work independent of MTOR. This important discovery revealed that MTOR is dispensable for autophagy induction. The authors showed that cAMP can modulate autophagy by controlling IP3 activity (Williams et al., 2008). As MTOR is central to several other pathways as well, identification of an alternative pathway opened the scope of controlling autophagy independent of MTOR.

### Luminescence Based High Throughput Assays

Luciferase being a sensitive reporter protein comes in handy when an assay has to be scaled to a high throughput format (Figure 1). Availability of different luciferase variants further helps in the design of an assay according to the needs. These luciferase variants have different degrees of sensitivity (Nanoluc is more sensitive to Firefly luciferase), different sizes and spectra (*Renilla* luciferase is smaller in size to Firefly luciferase) or different properties (Gaussian luciferase is secretory in nature while Renilla luciferase is cytosolic and Firefly luciferase naturally has a peroxisomal targeting signal). Depending on the need of the assay and the process to be studied, an appropriate luciferase variant may be used in the study. A *Gaussian* luciferase reporter based assay that quantitatively measures the autophagy rate by monitoring proteolytic activity of ATG4B, can be done at a large scale and is quantifiable (Ketteler and Seed, 2008). This luciferase release assay is well suited for upstream signaling events that either increase or decrease the rates of autophagy. A luciferase-based assay that exploited the property of long lived proteins to be solely degraded via autophagy pathway provided a direct relevance of the autophagy modulation in aggregate prone cells. This assay demonstrated autophagic clearance of an expanded polyglutamine *in vitro* and *in vivo* conditions (Ju et al., 2009). This assay takes into account the selective degradation of autophagy cargo using a sensitive luciferase-based reporter. Dynamic and sensitive assay could be achieved by following the cargo that is selective for degradation through autophagy. Peroxisomes provide highly inducible cargo with high turnover rates which are specifically degraded through autophagy machinery under starvation conditions (Sakai et al., 2006). This high turnover of peroxisomes when combined to the sensitivity of luciferase reporter, provides a very sensitive assay to monitor autophagic flux which is also amenable to high throughput setting. Based on this principle, Mishra et al., designed a screening strategy that allows measurement of autophagic cargo (facultative organelle, peroxisomes) clearance rather than ATG8 based changes in autophagosome number. The principle of the assay is based on detection of the levels of firefly and *Renilla* luciferase activities to monitor the flux of selective and general autophagy, respectively, in *S. cerevisiae* (Mishra et al., 2017b). Reporter strains were constructed that expressed Renilla luciferase and firefly luciferase with a peroxisome targeting signal (PTS1) under a fatty acid responsive promoter. These cells when grown in the presence of fatty acid or glycerol containing media, leads to the expression of peroxisomal firefly luciferase and *Renilla* luciferase which is cytosolic. These cells are then subjected to starvation to induce autophagy. Induction of autophagy leads to selective autophagic degradation of peroxisomes (pexophagy) and also non-selective bulk degradation of cytoplasm. The rate of decay in firefly luciferase activity depicts pexophagy whereas *Renilla* levels depict general autophagy. The dual luciferase assay provides the added advantage of monitoring autophagy in real time, is more sensitive and gives kinetic assessment of two different types of autophagy processes simultaneously. Interestingly, the action of the autophagy modulators identified from the screen was conserved across higher eukaryotes (Mishra et al., 2017a). The autophagy inhibitor Bay11 identified from the screen acted at the autophagosome biogenesis step and ZPCK inhibited the degradation of cargo inside the vacuole/lysosome. These inhibitors had a conserved mode of action across yeast, animals and plants.

Luciferase based HTS autophagy assay has been reported for mammalian cells as well. In a study by Min et al. (2018), a luciferase variant Luc2p was fused with the wild type p62/SQSTM1 or a deletion version of p62 (p62 lacking the ubiquitin binding domain) and transfected into glioma cells. The lysates from the two populations (wild type and mutant p62) were compared to monitor the autophagic flux. The performance of this probe was reported to be comparable to GFP-LC3-RFP-LC3ΔG probe described earlier in the review (Min et al., 2018).

### *In vitro* and *in silico* Assays

In recent years, many groups have also carried out a target driven autophagy screen using purified proteins and substrates. To identify substrates for ULK1 that might be involved in the process of autophagy, Egan et al. (2015), screened degenerate peptide libraries to identify a consensus motif for ULK1 mediated phosphorylation. After identifying novel phosphorylation sites, multiple targets for ULK1 were discovered. These substrates were then used to screen for potent inhibitors of ULK1 phosphorylation.

*Renilla* luciferase based turnover of LC3 was used to screen two kinase inhibitor libraries for identifying inhibitors of autophagic flux (Farkas et al., 2011). This study identified specific and more potent inhibitors of the upstream signaling component; class III phosphatidylinositol 3-kinase. Inhibitors specific to Ulk1 kinase activity, an upstream protein involved in autophagy initiation were obtained from a screen that utilized purified stress-activated Ulk1 and then looked at the phosphorylation of its substrate, Atg13 at Serine 318 position (Rosenberg et al., 2015).

Iorio et al. (2010) used the large dataset of drug expression pattern integrated into “drug network” and identified the previously hitherto unknown functions of several well characterized drugs. This is a dataset of expression profiles constructed while comparing the transcriptional responses induced by different small molecules in human cell lines. Through data mining, they identified fasudil as a novel autophagy enhancer taking the help of the same drug network (Iorio et al., 2010).

## Discussion

Although the core autophagy machinery and the proteins involved in disease conditions might be known, but the exact mechanism of action and how the autophagic flux is regulated is not completely understood which leads to many unanswered questions. Understanding and controlling the autophagic flux either through a genetic or pharmacological approach is a highly promising approach and of great scientific interest. Studies with genetic modulations of autophagic flux have been carried out in the past with immense success. Yoshinori Ohsumi, a pioneer in autophagy field was awarded the Nobel Prize in 2016 for his contribution to the study of autophagic flux. However, chemical modulation has an advantage over genetic manipulations that the phenotype could be observed just on the addition of the compound and the action could be reversed on its withdrawal. The method is less laborious, and the putative modulators could be used as leads for pharmacological purposes in certain disease conditions. However, there are limitations associated with the chemical approach because of the bioavailability issues, toxicity and the secondary or off-target effects associated with the chemical compound. Also, tissue specific effects are difficult to monitor.

To identify novel small molecule modulators of autophagy having a robust and sensitive screening system is the primary step. Therefore, HTS assays for autophagy are of utmost importance as they enable us to screen several small molecules in a small space of time with the inclusion of all possible biological and technical replicates. The data obtained from these assays should be amenable for direct comparison between the control and test groups and statistical analysis. Several high throughput assays have been developed in the recent past to identify small molecule modulators of autophagy. But some limitations associated with these assays must be overcome for a highly potent and effective HTS assay system. Many of these assays have issues with sensitivity and range. They do not directly look at the cargo or possess a higher physiological working range to detect smaller changes in autophagic flux. Although these assays are quantitative but may lack in one of the many parameters required to attain an ideal autophagy assay. An ideal assay would incorporate all these properties such as cargo build up, high sensitivity, ease of experimentation, broader physiological range, and live cell readout in a single high throughput format. Dynamic, sensitive and highly effective assay could be achieved by following the cargo that is inducible and selective for degradation through autophagy.

## Author Contributions

PM and RM conceived the idea and wrote the manuscript. VA conceptualized and contributed to the figure.

## Conflict of Interest Statement

The authors declare that the research was conducted in the absence of any commercial or financial relationships that could be construed as a potential conflict of interest.
